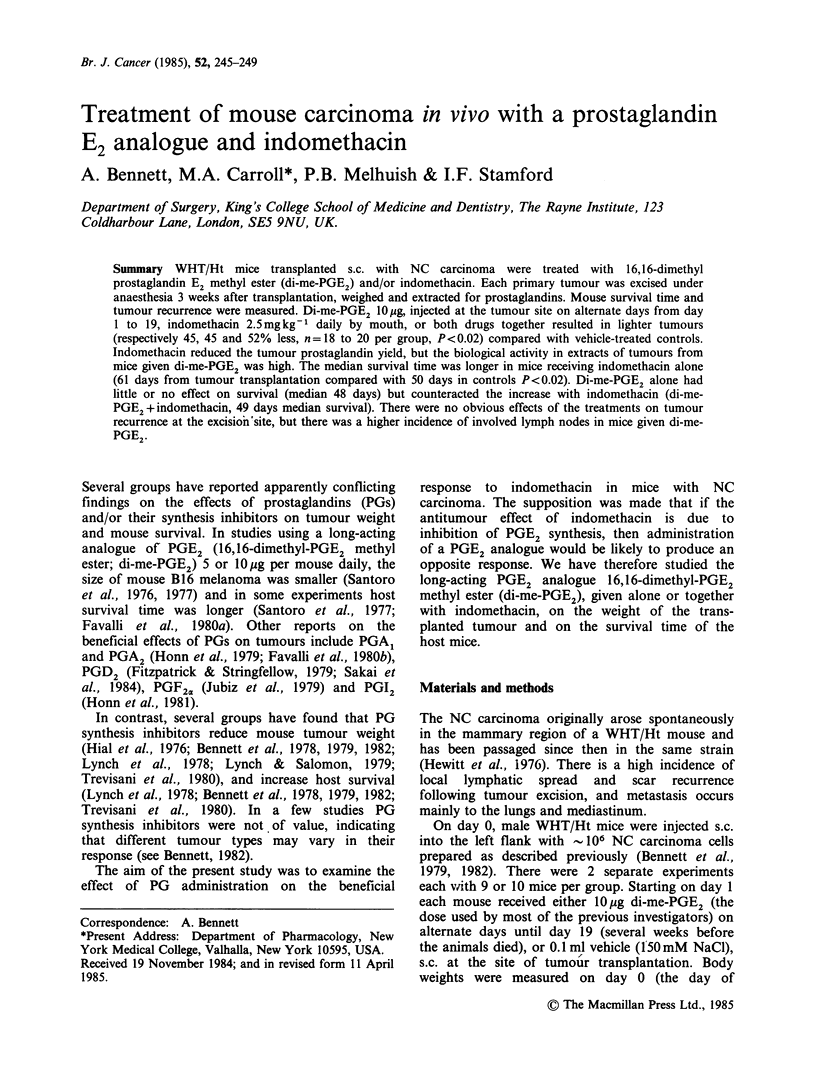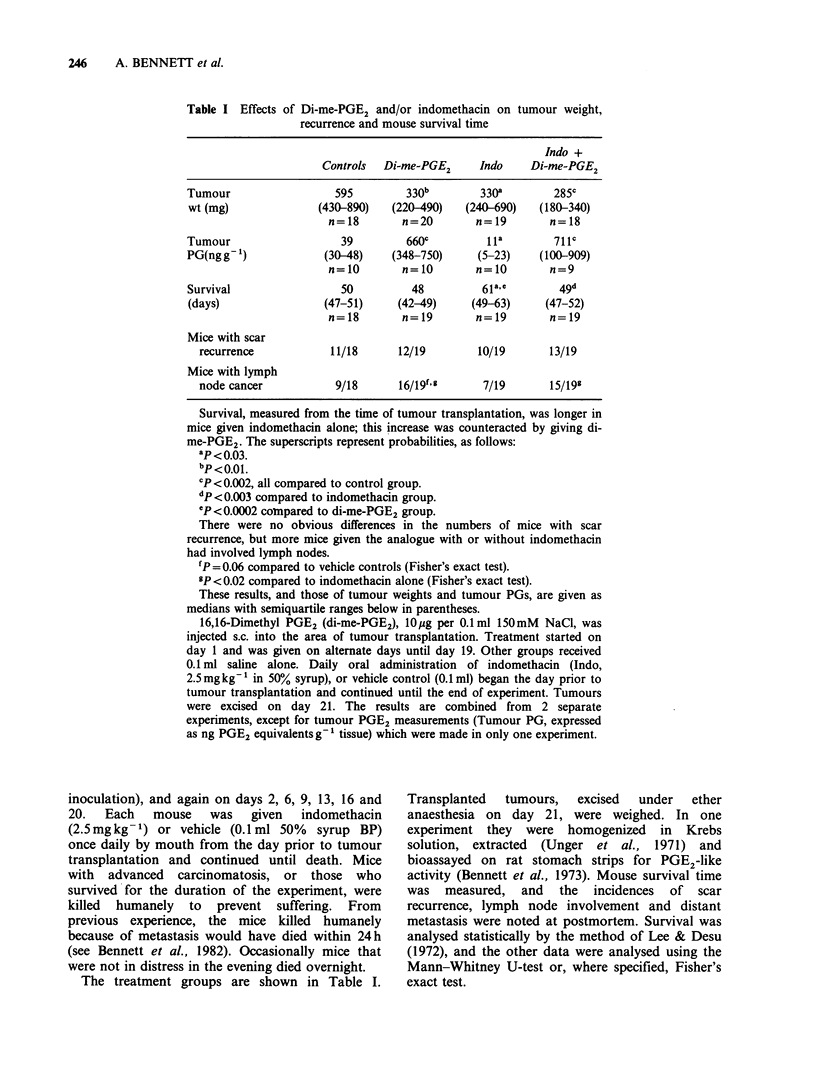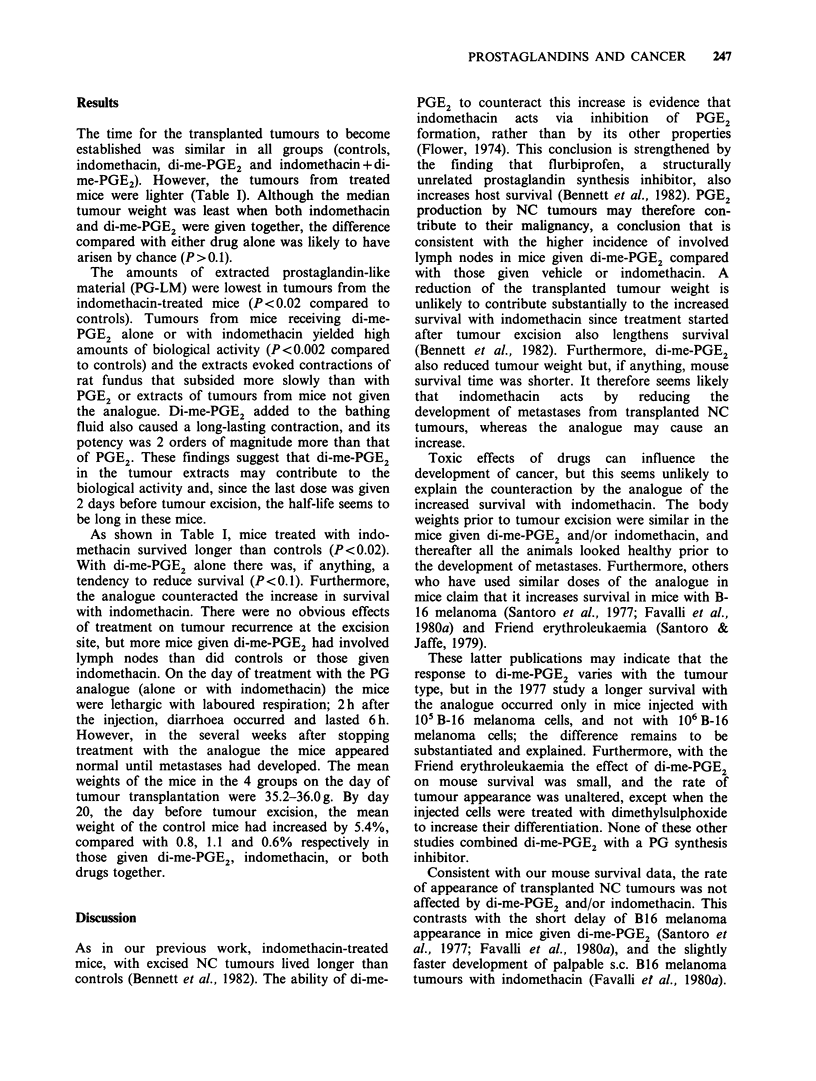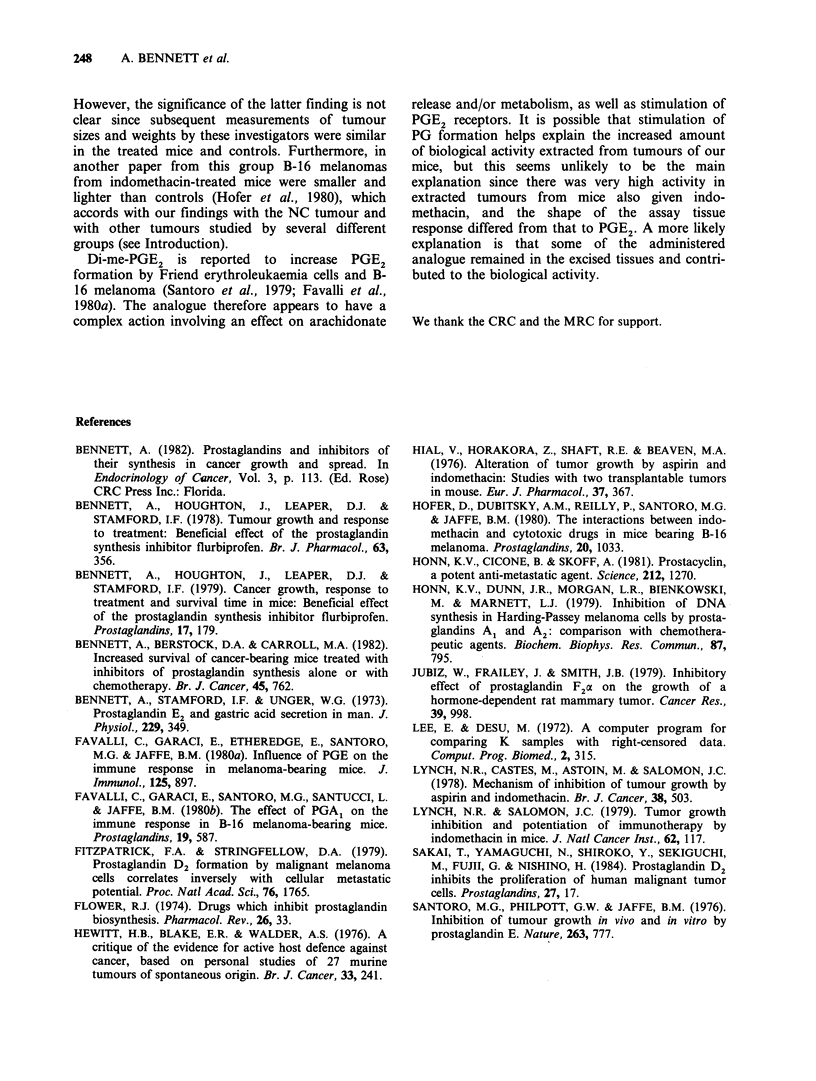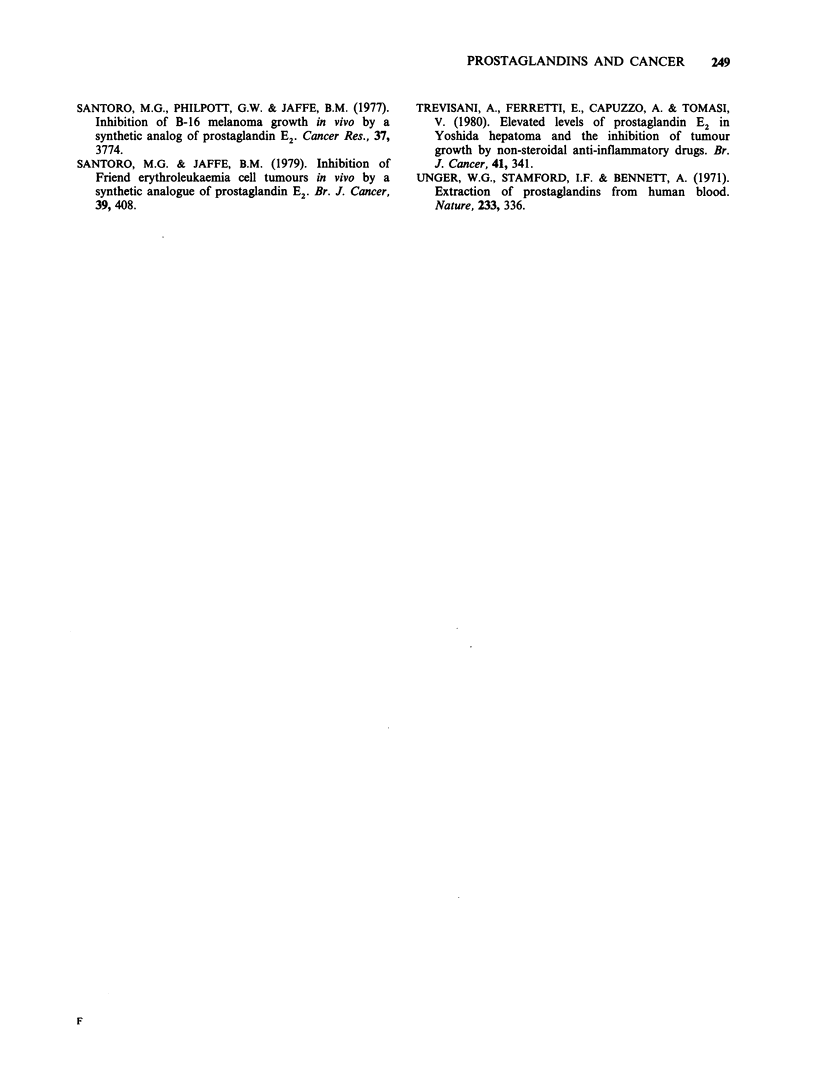# Treatment of mouse carcinoma in vivo with a prostaglandin E2 analogue and indomethacin.

**DOI:** 10.1038/bjc.1985.184

**Published:** 1985-08

**Authors:** A. Bennett, M. A. Carroll, P. B. Melhuish, I. F. Stamford

## Abstract

WHT/Ht mice transplanted s.c. with NC carcinoma were treated with 16,16-dimethyl prostaglandin E2 methyl ester (di-me-PGE2) and/or indomethacin. Each primary tumour was excised under anaesthesia 3 weeks after transplantation, weighed and extracted for prostaglandins. Mouse survival time and tumour recurrence were measured. Di-me-PGE2 10 micrograms, injected at the tumour site on alternate days from day 1 to 19, indomethacin 2.5 mg kg-1 daily by mouth, or both drugs together resulted in lighter tumours (respectively 45, 45 and 52% less, n = 18 to 20 per group, P less than 0.02) compared with vehicle-treated controls. Indomethacin reduced the tumour prostaglandin yield, but the biological activity in extracts of tumours from mice given di-me-PGE2 was high. The median survival time was longer in mice receiving indomethacin alone (61 days from tumour transplantation compared with 50 days in controls P less than 0.02). Di-me-PGE2 alone had little or no effect on survival (median 48 days) but counteracted the increase with indomethacin (di-me-PGE2 + indomethacin, 49 days median survival). There were no obvious effects of the treatments on tumour recurrence at the excision site, but there was a higher incidence of involved lymph nodes in mice given di-me-PGE2.


Br. J. Cancer (1985), 52, 245–249

### Treatment of mouse carcinoma *in vivo* with a prostaglandin $E_2$ analogue and indomethacin

A. Bennett, M.A. Carroll*, P.B. Melhuish & I.F. Stamford

*Department of Surgery, King's College School of Medicine and Dentistry, The Rayne Institute, 123 Coldharbour Lane, London, SE5 9NU, UK.*

**Summary** WHT/Ht mice transplanted s.c. with NC carcinoma were treated with 16,16-dimethyl prostaglandin $E_2$ methyl ester (di-me-$PGE_2$) and/or indomethacin. Each primary tumour was excised under anaesthesia 3 weeks after transplantation, weighed and extracted for prostaglandins. Mouse survival time and tumour recurrence were measured. Di-me-$PGE_2$ 10 $\mu g$, injected at the tumour site on alternate days from day 1 to 19, indomethacin 2.5 mg kg$^{-1}$ daily by mouth, or both drugs together resulted in lighter tumours (respectively 45, 45 and 52% less, $n=18$ to 20 per group, $P<0.02$) compared with vehicle-treated controls. Indomethacin reduced the tumour prostaglandin yield, but the biological activity in extracts of tumours from mice given di-me-$PGE_2$ was high. The median survival time was longer in mice receiving indomethacin alone (61 days from tumour transplantation compared with 50 days in controls $P<0.02$). Di-me-$PGE_2$ alone had little or no effect on survival (median 48 days) but counteracted the increase with indomethacin (di-me-$PGE_2$ + indomethacin, 49 days median survival). There were no obvious effects of the treatments on tumour recurrence at the excision site, but there was a higher incidence of involved lymph nodes in mice given di-me-$PGE_2$.

Several groups have reported apparently conflicting findings on the effects of prostaglandins (PGs) and/or their synthesis inhibitors on tumour weight and mouse survival. In studies using a long-acting analogue of $PGE_2$ (16,16-dimethyl-$PGE_2$ methyl ester; di-me-$PGE_2$) 5 or 10 $\mu g$ per mouse daily, the size of mouse B16 melanoma was smaller (Santoro *et al.*, 1976, 1977) and in some experiments host survival time was longer (Santoro *et al.*, 1977; Favalli *et al.*, 1980*a*). Other reports on the beneficial effects of PGs on tumours include $PGA_1$ and $PGA_2$ (Honn *et al.*, 1979; Favalli *et al.*, 1980*b*), $PGD_2$ (Fitzpatrick & Stringfellow, 1979; Sakai *et al.*, 1984), $PGF_{2\alpha}$ (Jubiz *et al.*, 1979) and $PGI_2$ (Honn *et al.*, 1981).

In contrast, several groups have found that PG synthesis inhibitors reduce mouse tumour weight (Hial *et al.*, 1976; Bennett *et al.*, 1978, 1979, 1982; Lynch *et al.*, 1978; Lynch & Salomon, 1979; Trevisani *et al.*, 1980), and increase host survival (Lynch *et al.*, 1978; Bennett *et al.*, 1978, 1979, 1982; Trevisani *et al.*, 1980). In a few studies PG synthesis inhibitors were not of value, indicating that different tumour types may vary in their response (see Bennett, 1982).

The aim of the present study was to examine the effect of PG administration on the beneficial response to indomethacin in mice with NC carcinoma. The supposition was made that if the antitumour effect of indomethacin is due to inhibition of $PGE_2$ synthesis, then administration of a $PGE_2$ analogue would be likely to produce an opposite response. We have therefore studied the long-acting $PGE_2$ analogue 16,16-dimethyl-$PGE_2$ methyl ester (di-me-$PGE_2$), given alone or together with indomethacin, on the weight of the transplanted tumour and on the survival time of the host mice.

### Materials and methods

The NC carcinoma originally arose spontaneously in the mammary region of a WHT/Ht mouse and has been passaged since then in the same strain (Hewitt *et al.*, 1976). There is a high incidence of local lymphatic spread and scar recurrence following tumour excision, and metastasis occurs mainly to the lungs and mediastinum.

On day 0, male WHT/Ht mice were injected s.c. into the left flank with $\sim 10^6$ NC carcinoma cells prepared as described previously (Bennett *et al.*, 1979, 1982). There were 2 separate experiments each with 9 or 10 mice per group. Starting on day 1 each mouse received either 10 $\mu g$ di-me-$PGE_2$ (the dose used by most of the previous investigators) on alternate days until day 19 (several weeks before the animals died), or 0.1 ml vehicle (150 mM NaCl), s.c. at the site of tumour transplantation. Body weights were measured on day 0 (the day of

Correspondence: A. Bennett
*Present Address: Department of Pharmacology, New York Medical College, Valhalla, New York 10595, USA.
Received 19 November 1984; and in revised form 11 April 1985.

© The Macmillan Press Ltd., 1985

**Table I** Effects of Di-me-$PGE_2$ and/or indomethacin on tumour weight, recurrence and mouse survival time

| | *Controls* | *Di-me-$PGE_2$* | *Indo* | *Indo + Di-me-$PGE_2$* |
|---|---|---|---|---|
| Tumour wt (mg) | 595 (430–890) $n=18$ | 330[b] (220–490) $n=20$ | 330[a] (240–690) $n=19$ | 285[c] (180–340) $n=18$ |
| Tumour PG($ng\,g^{-1}$) | 39 (30–48) $n=10$ | 660[c] (348–750) $n=10$ | 11[a] (5–23) $n=10$ | 711[c] (100–909) $n=9$ |
| Survival (days) | 50 (47–51) $n=18$ | 48 (42–49) $n=19$ | 61[a,e] (49–63) $n=19$ | 49[d] (47–52) $n=19$ |
| Mice with scar recurrence | 11/18 | 12/19 | 10/19 | 13/19 |
| Mice with lymph node cancer | 9/18 | 16/19[f,g] | 7/19 | 15/19[g] |

Survival, measured from the time of tumour transplantation, was longer in mice given indomethacin alone; this increase was counteracted by giving di-me-$PGE_2$. The superscripts represent probabilities, as follows:
[a]$P<0.03$.
[b]$P<0.01$.
[c]$P<0.002$, all compared to control group.
[d]$P<0.003$ compared to indomethacin group.
[e]$P<0.0002$ compared to di-me-$PGE_2$ group.
There were no obvious differences in the numbers of mice with scar recurrence, but more mice given the analogue with or without indomethacin had involved lymph nodes.
[f]$P=0.06$ compared to vehicle controls (Fisher's exact test).
[g]$P<0.02$ compared to indomethacin alone (Fisher's exact test).
These results, and those of tumour weights and tumour PGs, are given as medians with semiquartile ranges below in parentheses.
16,16-Dimethyl $PGE_2$ (di-me-$PGE_2$), 10 $\mu g$ per 0.1 ml 150 mM NaCl, was injected s.c. into the area of tumour transplantation. Treatment started on day 1 and was given on alternate days until day 19. Other groups received 0.1 ml saline alone. Daily oral administration of indomethacin (Indo, 2.5 $mg\,kg^{-1}$ in 50% syrup), or vehicle control (0.1 ml) began the day prior to tumour transplantation and continued until the end of experiment. Tumours were excised on day 21. The results are combined from 2 separate experiments, except for tumour $PGE_2$ measurements (Tumour PG, expressed as ng $PGE_2$ equivalents $g^{-1}$ tissue) which were made in only one experiment.

inoculation), and again on days 2, 6, 9, 13, 16 and 20. Each mouse was given indomethacin (2.5 $mg\,kg^{-1}$) or vehicle (0.1 ml 50% syrup BP) once daily by mouth from the day prior to tumour transplantation and continued until death. Mice with advanced carcinomatosis, or those who survived for the duration of the experiment, were killed humanely to prevent suffering. From previous experience, the mice killed humanely because of metastasis would have died within 24 h (see Bennett *et al.*, 1982). Occasionally mice that were not in distress in the evening died overnight.

The treatment groups are shown in Table I. Transplanted tumours, excised under ether anaesthesia on day 21, were weighed. In one experiment they were homogenized in Krebs solution, extracted (Unger *et al.*, 1971) and bioassayed on rat stomach strips for $PGE_2$-like activity (Bennett *et al.*, 1973). Mouse survival time was measured, and the incidences of scar recurrence, lymph node involvement and distant metastasis were noted at postmortem. Survival was analysed statistically by the method of Lee & Desu (1972), and the other data were analysed using the Mann–Whitney U-test or, where specified, Fisher's exact test.

### Results

The time for the transplanted tumours to become established was similar in all groups (controls, indomethacin, di-me-$PGE_2$ and indomethacin + di-me-$PGE_2$). However, the tumours from treated mice were lighter (Table I). Although the median tumour weight was least when both indomethacin and di-me-$PGE_2$ were given together, the difference compared with either drug alone was likely to have arisen by chance ($P > 0.1$).

The amounts of extracted prostaglandin-like material (PG-LM) were lowest in tumours from the indomethacin-treated mice ($P < 0.02$ compared to controls). Tumours from mice receiving di-me-$PGE_2$ alone or with indomethacin yielded high amounts of biological activity ($P < 0.002$ compared to controls) and the extracts evoked contractions of rat fundus that subsided more slowly than with $PGE_2$ or extracts of tumours from mice not given the analogue. Di-me-$PGE_2$ added to the bathing fluid also caused a long-lasting contraction, and its potency was 2 orders of magnitude more than that of $PGE_2$. These findings suggest that di-me-$PGE_2$ in the tumour extracts may contribute to the biological activity and, since the last dose was given 2 days before tumour excision, the half-life seems to be long in these mice.

As shown in Table I, mice treated with indomethacin survived longer than controls ($P < 0.02$). With di-me-$PGE_2$ alone there was, if anything, a tendency to reduce survival ($P < 0.1$). Furthermore, the analogue counteracted the increase in survival with indomethacin. There were no obvious effects of treatment on tumour recurrence at the excision site, but more mice given di-me-$PGE_2$ had involved lymph nodes than did controls or those given indomethacin. On the day of treatment with the PG analogue (alone or with indomethacin) the mice were lethargic with laboured respiration; 2 h after the injection, diarrhoea occurred and lasted 6 h. However, in the several weeks after stopping treatment with the analogue the mice appeared normal until metastases had developed. The mean weights of the mice in the 4 groups on the day of tumour transplantation were 35.2–36.0 g. By day 20, the day before tumour excision, the mean weight of the control mice had increased by 5.4%, compared with 0.8, 1.1 and 0.6% respectively in those given di-me-$PGE_2$, indomethacin, or both drugs together.

### Discussion

As in our previous work, indomethacin-treated mice, with excised NC tumours lived longer than controls (Bennett *et al.*, 1982). The ability of di-me-$PGE_2$ to counteract this increase is evidence that indomethacin acts via inhibition of $PGE_2$ formation, rather than by its other properties (Flower, 1974). This conclusion is strengthened by the finding that flurbiprofen, a structurally unrelated prostaglandin synthesis inhibitor, also increases host survival (Bennett *et al.*, 1982). $PGE_2$ production by NC tumours may therefore contribute to their malignancy, a conclusion that is consistent with the higher incidence of involved lymph nodes in mice given di-me-$PGE_2$ compared with those given vehicle or indomethacin. A reduction of the transplanted tumour weight is unlikely to contribute substantially to the increased survival with indomethacin since treatment started after tumour excision also lengthens survival (Bennett *et al.*, 1982). Furthermore, di-me-$PGE_2$ also reduced tumour weight but, if anything, mouse survival time was shorter. It therefore seems likely that indomethacin acts by reducing the development of metastases from transplanted NC tumours, whereas the analogue may cause an increase.

Toxic effects of drugs can influence the development of cancer, but this seems unlikely to explain the counteraction by the analogue of the increased survival with indomethacin. The body weights prior to tumour excision were similar in the mice given di-me-$PGE_2$ and/or indomethacin, and thereafter all the animals looked healthy prior to the development of metastases. Furthermore, others who have used similar doses of the analogue in mice claim that it increases survival in mice with B-16 melanoma (Santoro *et al.*, 1977; Favalli *et al.*, 1980*a*) and Friend erythroleukaemia (Santoro & Jaffe, 1979).

These latter publications may indicate that the response to di-me-$PGE_2$ varies with the tumour type, but in the 1977 study a longer survival with the analogue occurred only in mice injected with $10^5$ B-16 melanoma cells, and not with $10^6$ B-16 melanoma cells; the difference remains to be substantiated and explained. Furthermore, with the Friend erythroleukaemia the effect of di-me-$PGE_2$ on mouse survival was small, and the rate of tumour appearance was unaltered, except when the injected cells were treated with dimethylsulphoxide to increase their differentiation. None of these other studies combined di-me-$PGE_2$ with a PG synthesis inhibitor.

Consistent with our mouse survival data, the rate of appearance of transplanted NC tumours was not affected by di-me-$PGE_2$ and/or indomethacin. This contrasts with the short delay of B16 melanoma appearance in mice given di-me-$PGE_2$ (Santoro *et al.*, 1977; Favalli *et al.*, 1980*a*), and the slightly faster development of palpable s.c. B16 melanoma tumours with indomethacin (Favalli *et al.*, 1980*a*).

However, the significance of the latter finding is not clear since subsequent measurements of tumour sizes and weights by these investigators were similar in the treated mice and controls. Furthermore, in another paper from this group B-16 melanomas from indomethacin-treated mice were smaller and lighter than controls (Hofer *et al.*, 1980), which accords with our findings with the NC tumour and with other tumours studied by several different groups (see Introduction).

Di-me-$PGE_2$ is reported to increase $PGE_2$ formation by Friend erythroleukaemia cells and B-16 melanoma (Santoro *et al.*, 1979; Favalli *et al.*, 1980*a*). The analogue therefore appears to have a complex action involving an effect on arachidonate release and/or metabolism, as well as stimulation of $PGE_2$ receptors. It is possible that stimulation of PG formation helps explain the increased amount of biological activity extracted from tumours of our mice, but this seems unlikely to be the main explanation since there was very high activity in extracted tumours from mice also given indomethacin, and the shape of the assay tissue response differed from that to $PGE_2$. A more likely explanation is that some of the administered analogue remained in the excised tissues and contributed to the biological activity.

We thank the CRC and the MRC for support.

### References

BENNETT, A. (1982). Prostaglandins and inhibitors of their synthesis in cancer growth and spread. In *Endocrinology of Cancer*, Vol. 3, p. 113. (Ed. Rose) CRC Press Inc.: Florida.

BENNETT, A., HOUGHTON, J., LEAPER, D.J. & STAMFORD, I.F. (1978). Tumour growth and response to treatment: Beneficial effect of the prostaglandin synthesis inhibitor flurbiprofen. *Br. J. Pharmacol.*, **63**, 356.

BENNETT, A., HOUGHTON, J., LEAPER, D.J. & STAMFORD, I.F. (1979). Cancer growth, response to treatment and survival time in mice: Beneficial effect of the prostaglandin synthesis inhibitor flurbiprofen. *Prostaglandins*, **17**, 179.

BENNETT, A., BERSTOCK, D.A. & CARROLL, M.A. (1982). Increased survival of cancer-bearing mice treated with inhibitors of prostaglandin synthesis alone or with chemotherapy. *Br. J. Cancer*, **45**, 762.

BENNETT, A., STAMFORD, I.F. & UNGER, W.G. (1973). Prostaglandin $E_2$ and gastric acid secretion in man. *J. Physiol.*, **229**, 349.

FAVALLI, C., GARACI, E., ETHEREDGE, E., SANTORO, M.G. & JAFFE, B.M. (1980*a*). Influence of PGE on the immune response in melanoma-bearing mice. *J. Immunol.*, **125**, 897.

FAVALLI, C., GARACI, E., SANTORO, M.G., SANTUCCI, L. & JAFFE, B.M. (1980*b*). The effect of $PGA_1$ on the immune response in B-16 melanoma-bearing mice. *Prostaglandins*, **19**, 587.

FITZPATRICK, F.A. & STRINGFELLOW, D.A. (1979). Prostaglandin $D_2$ formation by malignant melanoma cells correlates inversely with cellular metastatic potential. *Proc. Natl Acad. Sci.*, **76**, 1765.

FLOWER, R.J. (1974). Drugs which inhibit prostaglandin biosynthesis. *Pharmacol. Rev.*, **26**, 33.

HEWITT, H.B., BLAKE, E.R. & WALDER, A.S. (1976). A critique of the evidence for active host defence against cancer, based on personal studies of 27 murine tumours of spontaneous origin. *Br. J. Cancer*, **33**, 241.

HIAL, V., HORAKORA, Z., SHAFT, R.E. & BEAVEN, M.A. (1976). Alteration of tumor growth by aspirin and indomethacin: Studies with two transplantable tumors in mouse. *Eur. J. Pharmacol.*, **37**, 367.

HOFER, D., DUBITSKY, A.M., REILLY, P., SANTORO, M.G. & JAFFE, B.M. (1980). The interactions between indomethacin and cytotoxic drugs in mice bearing B-16 melanoma. *Prostaglandins*, **20**, 1033.

HONN, K.V., CICONE, B. & SKOFF, A. (1981). Prostacyclin, a potent anti-metastatic agent. *Science*, **212**, 1270.

HONN, K.V., DUNN, J.R., MORGAN, L.R., BIENKOWSKI, M. & MARNETT, L.J. (1979). Inhibition of DNA synthesis in Harding-Passey melanoma cells by prostaglandins $A_1$ and $A_2$: comparison with chemotherapeutic agents. *Biochem. Biophys. Res. Commun.*, **87**, 795.

JUBIZ, W., FRAILEY, J. & SMITH, J.B. (1979). Inhibitory effect of prostaglandin $F_2\alpha$ on the growth of a hormone-dependent rat mammary tumor. *Cancer Res.*, **39**, 998.

LEE, E. & DESU, M. (1972). A computer program for comparing K samples with right-censored data. *Comput. Prog. Biomed.*, **2**, 315.

LYNCH, N.R., CASTES, M., ASTOIN, M. & SALOMON, J.C. (1978). Mechanism of inhibition of tumour growth by aspirin and indomethacin. *Br. J. Cancer*, **38**, 503.

LYNCH, N.R. & SALOMON, J.C. (1979). Tumor growth inhibition and potentiation of immunotherapy by indomethacin in mice. *J. Natl Cancer Inst.*, **62**, 117.

SAKAI, T., YAMAGUCHI, N., SHIROKO, Y., SEKIGUCHI, M., FUJII, G. & NISHINO, H. (1984). Prostaglandin $D_2$ inhibits the proliferation of human malignant tumor cells. *Prostaglandins*, **27**, 17.

SANTORO, M.G., PHILPOTT, G.W. & JAFFE, B.M. (1976). Inhibition of tumour growth *in vivo* and *in vitro* by prostaglandin E. *Nature*, **263**, 777.

SANTORO, M.G., PHILPOTT, G.W. & JAFFE, B.M. (1977). Inhibition of B-16 melanoma growth *in vivo* by a synthetic analog of prostaglandin $E_2$. *Cancer Res.*, **37**, 3774.

SANTORO, M.G. & JAFFE, B.M. (1979). Inhibition of Friend erythroleukaemia cell tumours *in vivo* by a synthetic analogue of prostaglandin $E_2$. *Br. J. Cancer*, **39**, 408.

TREVISANI, A., FERRETTI, E., CAPUZZO, A. & TOMASI, V. (1980). Elevated levels of prostaglandin $E_2$ in Yoshida hepatoma and the inhibition of tumour growth by non-steroidal anti-inflammatory drugs. *Br. J. Cancer*, **41**, 341.

UNGER, W.G., STAMFORD, I.F. & BENNETT, A. (1971). Extraction of prostaglandins from human blood. *Nature*, **233**, 336.